# Supplementary material for: Targeting mycobacterial transpeptidases: evaluating the roles of Ldt and PBP inhibition in suppressing Mycobacterium smegmatis
Source: Antimicrob Agents Chemother. 2025 Sep 15;69(10):e00126-25. doi: 10.1128/aac.00126-25 (PMC12486854; doi:10.1128/aac.00126-25)
Supplement: Supplemental material — Tables S1 to S6; Fig. S1 to S13. [file aac.00126-25-s0001.pdf]

Supplementary information

Targeting Mycobacterial Transpeptidases: Evaluating the Roles of Ldt and PBP Inhibition in Suppressing *Mycobacterium smegmatis*

Mariska de Munnik,[1] Karina Calvopiña,[1] Patrick Rabe,[1,2] and Christopher J. Schofield[1*]

[1] Chemistry Research Laboratory, Department of Chemistry and the Ineos Oxford Institute of Antimicrobial Research, University of Oxford, 12 Mansfield Road, Oxford, OX1 3TA, United Kingdom.

[2] Diamond Light Source, Diamond House, Harwell Science and Innovation Campus, Didcot OX11 0DE, UK.

* Email: christopher.schofield@chem.ox.ac.uk

Table S1. Activity of β-lactams and Ldt_{Mt2} inhibitors against *Mycobacterium smegmatis*. MIC values represent the lowest concentration in which no bacterial growth was observed, as determined by OD_{600} measurement and resazurin staining. The *Msm* MIC values were determined with three independent repeats. The inhibition of *Msm* PBPs (pIC_{50} *Msm* PBP) and Ldts (pIC_{50} *Msm* PBP) was determined using the fluorescent peptide incorporation method in duplicate assays.(1) Inhibition of recombinant *Mtb* PBP3 (pIC_{50} values for *Mtb* PBP3) was determined in quadruplicate assays. For reference, reported inhibition results recombinant Ldt_{Mt2} (pIC_{50} values for Ldt_{Mt2}) are shown.(2, 3) Errors represent standard deviation from the mean. N.D. represents not determined. CLAV = clavulanic acid. See methods for experimental details.

**Compound**	**Structure**	**MIC *Msm* (µg/mL)**		**pIC_{50} *Msm* PBP (mean ± SD)**	**pIC_{50} *Msm* Ldt (mean ± SD)**	**pIC_{50} *Mtb* PBP3 (mean ± SD)**	**pIC_{50} Ldt_{Mt2} (mean ± SD)**
		**No CLAV**	**CLAV (100 µg/mL)**				
**1** Faropenem	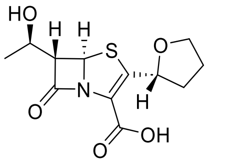	16	1	6.3 ± 0.051	5.1 ± 0.032	6.5 ± 0.042	6.5 ± 0.042
**2** Meropenem	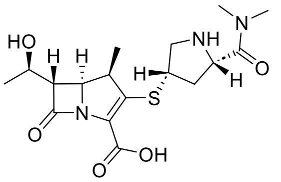	2	<0.063	6.6 ± 0.055	5.5 ± 0.029	6.6 ± 0.029	5.0 ± 0.1
**3** Imipenem	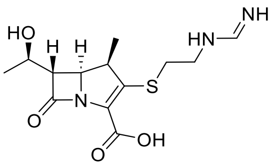	0.125	<0.063	9.2 ± 0.054	7.5 ± 0.047	6.3 ± 0.069	6.1 ± 0.1
**4** Ertapenem	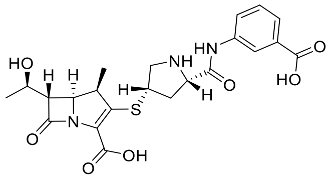	4	2	5.7 ± 0.14	5.4 ± 0.092	6.7 ± 0.032	6.1 ± 0.1
**5** Doripenem	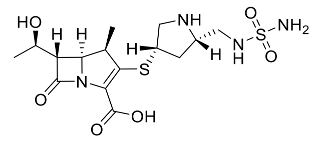	0.5	<0.063	6.2 ± 0.070	6.1 ± 0.13	6.6 ± 0.031	4.9 ± 0.1
**6** Ceftazidime	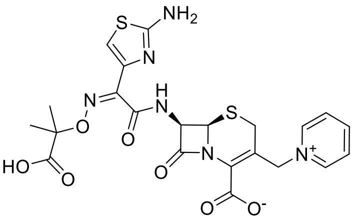	>128	128	4.1 ± 0.069	3.8 ± 0.69	5.5 ± 0.040	<3.4

Compound	Structure	MIC *Msm* (μg/mL)		pIC_{50} *Msm* PBP (mean ± SD)	pIC_{50} *Msm* Ldt (mean ± SD)	pIC_{50} *Mtb* PBP3 (mean ± SD)	pIC_{50} Ldt_{Mt2} (mean ± SD)
		No CLAV	CLAV (100 μg/mL)				
**7** Ceftriaxone	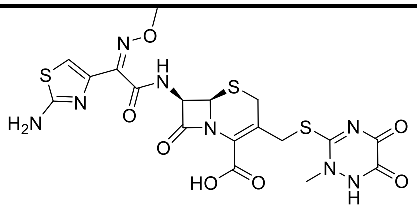	>128	128	4.5 ± 0.057	4.3 ± 0.10	6.6 ± 0.22	<3.4
**8** Cephalothin	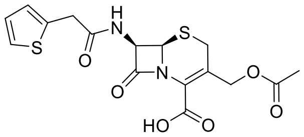	N.D.	2	4.3 ± 0.10	4.1 ± 0.091	6.6 ± 0.11	<3.4
**9** Cefmetazole	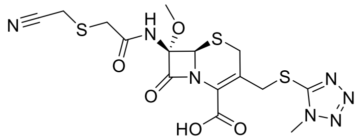	N.D.	4	4.2 ± 0.38	4.8 ± 0.13	6.6 ± 0.088	N.D.
**10** Cefepime	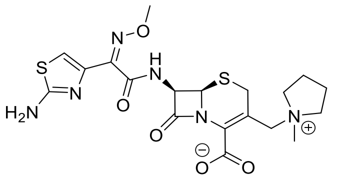	N.D.	128	5.2 ± 0.11	4.2 ± 0.11	6.5 ± 0.042	<3.4
**11** Ampicillin	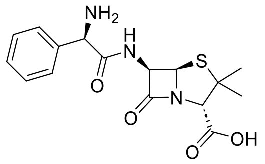	>128	2	6.2 ± 0.056	4.7 ± 0.079	6.6 ± 0.031	<3.4
**12** Amoxicillin	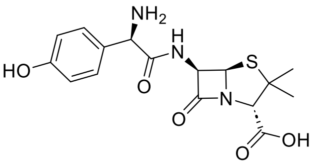	>128	1	5.8 ± 0.11	4.4 ± 0.071	6.9 ± 0.27	<3.4
**13** Oxacillin	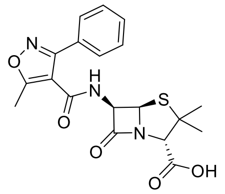	>128	>128	<3.6	<3.6	6.6 ± 0.045	5.2 ±.0.1
**14** Penicillin G	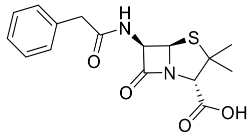	N.D.	32	5.3 ± 0.070	<3.6	6.7 ± 0.046	3.7 ± 0.1
**15** Carbenicillin	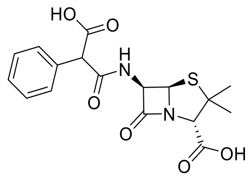	N.D.	64	5.2 ± 0.042	<3.6	5.4 ± 0.035	<3.4

Compound	Structure	MIC *Msm* (μg/mL)		pIC$_{50}$ *Msm* PBP (mean ± SD)	pIC$_{50}$ *Msm* Ldt (mean ± SD)	pIC$_{50}$ *Mtb* PBP3 (mean ± SD)	pIC$_{50}$ Ldt$_{Mt2}$ (mean ± SD)
		No CLAV	CLAV (100 μg/mL)				
**16** Aztreonam	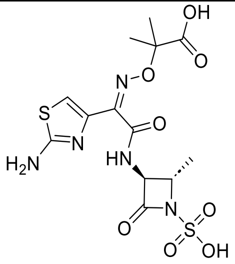	>128	>128	<3.6	<3.6	3.8 ± 0.16	<3.4
**17** Clavulanic acid	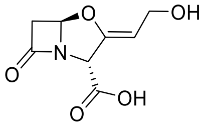	128	N.D.	4.3 ± 0.14	<3.6	<3.4	3.7 ± 0.1
**18**	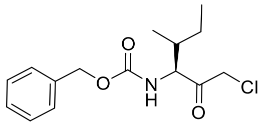	>128	N.D.	N.D.	N.D.	<3.4	7.1 ± 0.045
**19**	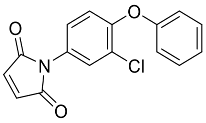	64	N.D.	N.D.	N.D.	4.0 ± 0.15	7.4 ± 0.010
**20**	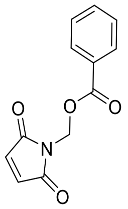	64	N.D.	N.D.	N.D.	4.5 ± 0.20	7.0 ± 0.00
**21**	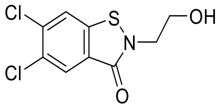	32	N.D.	4.6 ± 0.080	4.4 ± 0.063	4.1 ± 0.15	8.0 ± 0.040
**22**	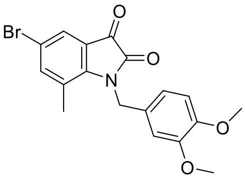	>128	N.D.	N.D.	N.D.	<3.4	6.2 ± 0.14
**23**	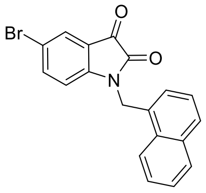	>128	N.D.	N.D.	N.D.	N.D.	6.5 ± 0.005

**Compound**	**Structure**	**MIC *Msm* (µg/mL)**		**pIC_{50} *Msm* PBP (mean ± SD)**	**pIC_{50} *Msm* Ldt (mean ± SD)**	**pIC_{50} *Mtb* PBP3 (mean ± SD)**	**pIC_{50} Ldt_{Mt2} (mean ± SD)**
		**No CLAV**	**CLAV (100 µg/mL)**				
**24**	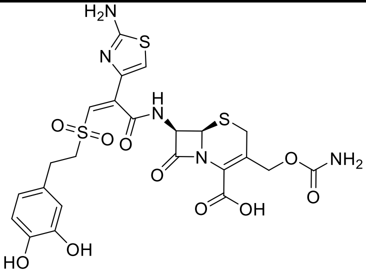	64	N.D.	3.7 ± 0.25	3.8 ± 0.090	6.1 ± 0.089	5.6 ± 0.045
**25**	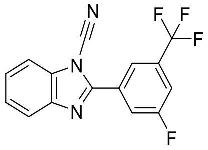	48	N.D.	4.5 ± 0.10	4.8 ± 0.047	<3.4	7.3 ± 0.065
**26**	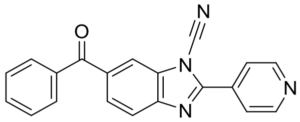	16	N.D.	N.D.	N.D.	4.5 ± 0.085	7.1 ± 0.050
**27**	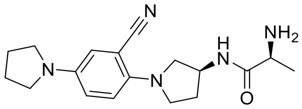	64	N.D.	N.D.	N.D.	N.D.	6.3 ± 0.065
**28**	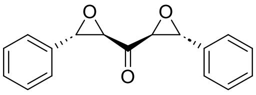	>128	N.D.	N.D.	N.D.	<3.4	7.2 ± 0.012
**29**	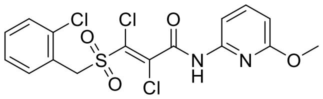	>128	N.D.	N.D.	N.D.	<3.4	7.2 ± 0.035
**30**	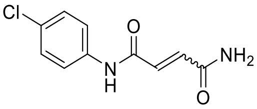	>128	N.D.	N.D.	N.D.	<3.4	6.4 ± 0.080
**31**	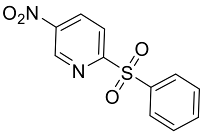	8	N.D.	<3.6	4.2 ± 0.071	<3.4	5.3 ± 0.1

Table S2. Activity of cephalothin against *Mycobacterium smegmatis* in the presence of β-lactamase inhibitors. MIC experiments were performed in triplicate. CEP = cephalothin, BLI = β-lactamase inhibitor.

Compound	Structure	MIC (µg/mL) CEP + 0 µg/mL BLI	MIC (µg/mL) CEP + 10 µg/mL BLI	MIC (µg/mL) CEP + 50 µg/mL BLI	MIC (µg/mL) CEP + 100 µg/mL BLI
**17** Clavulanic acid	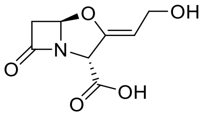	>128	64	16	2
Tazobactam	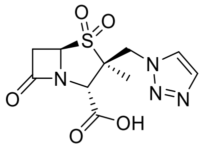	>128	>128	128	32
Sulbactam	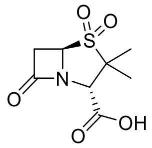	>128	>128	>128	>128
BLI-489	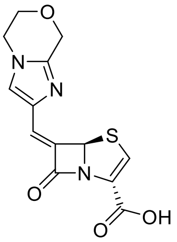	>128	>128	32	8
Xeruborbactam	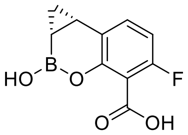	>128	>128	>128	>128
Avibactam	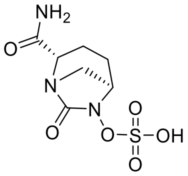	>128	>128	>128	>128

Table S3. Activities of control compounds ethambutol, isoniazid, and rifampicin against *Mycobacterium smegmatis.* MIC experiments were performed in triplicate. For reference, reported MICs for ethambutol, isoniazid, and rifampicin activities against *Msm* are provided.(4, 5)

**Compound**	**MIC (μg/mL)**	**Reported MIC (μg/mL)** (4, 5)
Ethambutol	0.25	0.25 – 5
Isoniazid	4 – 8	0.2 – 10
Rifampicin	2 – 4	0.8 – 1.6

Table S4. MIC studies of treatment of *Mycobacterium smegmatis* with Ldt_{Mt2} inhibitors combined with faropenem or meropenem. MIC values were determined using three independent assays. Values in blue represent inhibitor combinations that indicate an improved activity compared to single dosing. Hyphens indicate concentration of inhibitor combinations that are above the MIC of the Ldt_{Mt2} inhibitor alone (Table S1). Structures of the Ldt_{Mt2} inhibitors are given in Table S1. FAR = faropenem, MER = meropenem.

Ldt_{Mt2} inhibitor	MIC (µg/mL)					
	FAR + 64 µg/mL Ldt_{Mt2} inhibitor	FAR + 16 µg/mL Ldt_{Mt2} inhibitor]	FAR + 4 µg/mL Ldt_{Mt2} inhibitor	MER + 64 µg/mL Ldt_{Mt2} inhibitor	MER + 16 µg/mL Ldt_{Mt2} inhibitor]	MER + 4 µg/mL Ldt_{Mt2} inhibitor
**19**	-	16	16	-	2	2
**21**	-	<0.25	8	-	<0.25	2
**24**	-	16	16	-	2	2
**25**	-	1	4	-	1	2
**26**	-	-	8	-	-	2
**29**	16	16	16	2	2	2
**30**	16	16	16	2	2	2
**31**	-	-	<0.25	-	-	<0.25

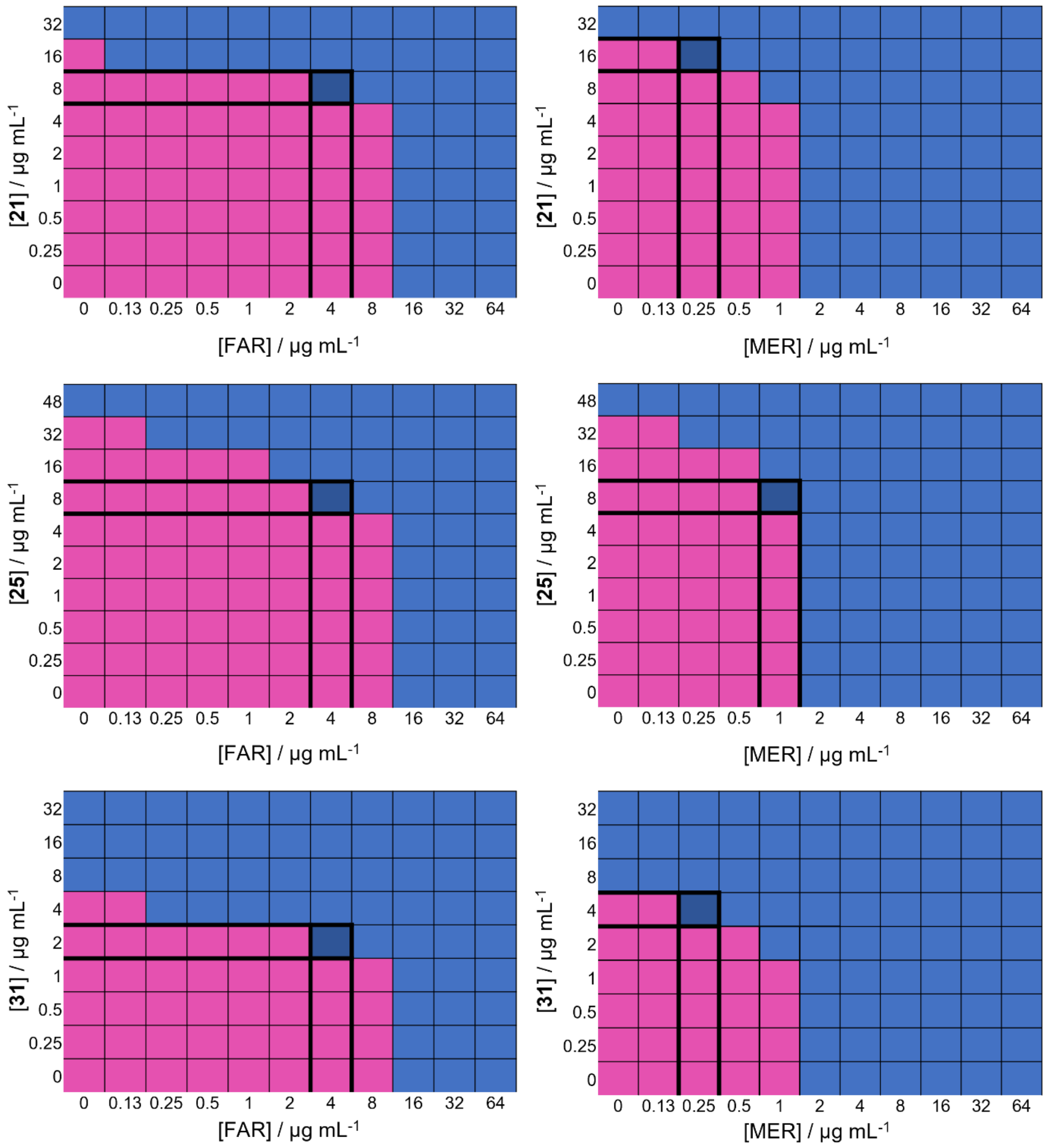

Figure S1. Activities of 21, 25, or 31, in combination with faropenem or meropenem against *Mycobacterium smegmatis*. Inhibitor concentrations that resulted in bacterial growth are in pink. Inhibitor concentrations that resulted in no evidence for bacterial growth are in blue. Inhibitor concentrations leading to optimal synergistic and/or additive effects are in a darker shade of blue, and bordered by black lines. Experiments were performed in triplicate. FAR = faropenem, MER = meropenem.

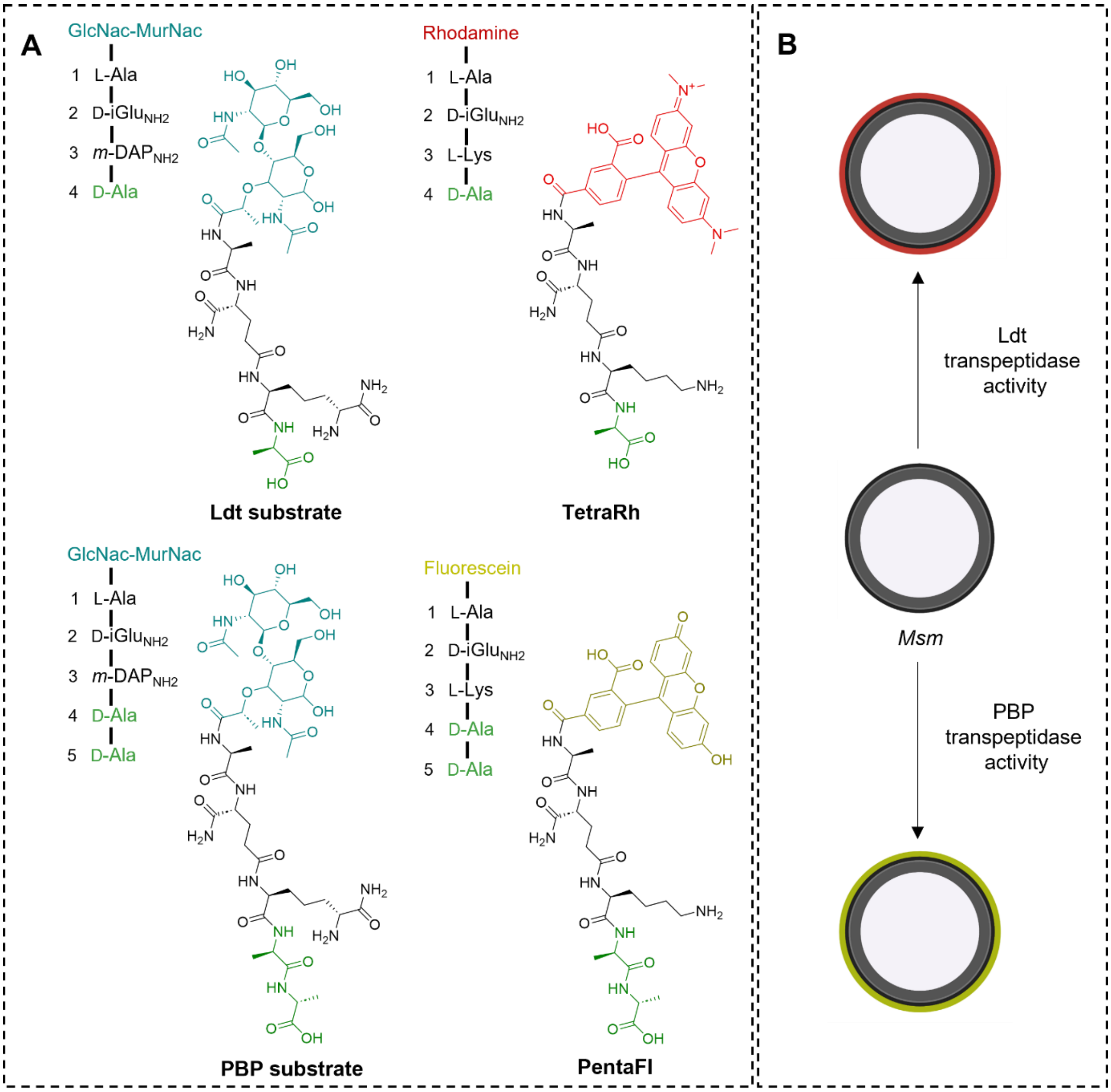

Figure S2. Principle of the fluorescent peptide incorporation assays. A. TetraRh and PentaFl, as reported by Pidgeon *et al.* (2019)(1) are fluorescent derivatives of the Ldt and PBP substrate monomers, respectively. **B.** Incubation of *Msm* with TetraRh and PentaFl leads to their incorporation into the cell wall, dependent on Ldt and PBP activities, respectively.

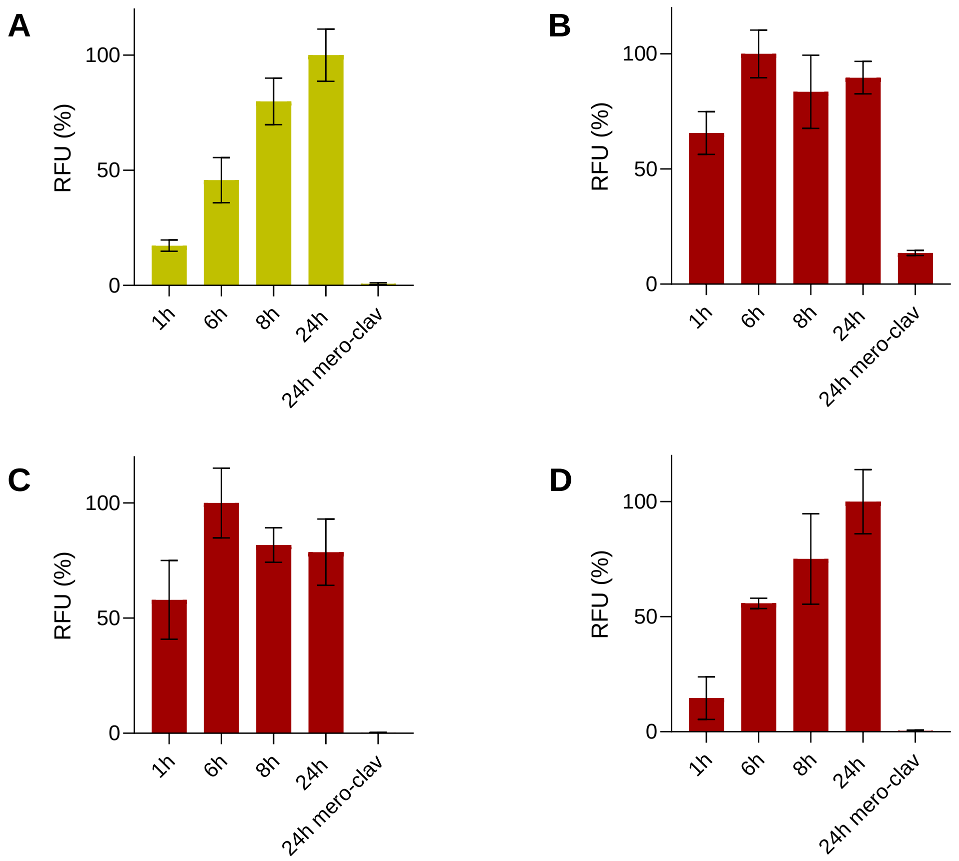

Figure S3. Time dependent incorporation of PentaFl (A) or TetraRh (B-D) into *Mycobacterium smegmatis*. A culture of *Msm* (OD_{600} 0.8) was incubated with 50 µM PentaFl (**A**), or 50 µM (**B**), 5 µM (**C**), or 0.5 µM (**D**) TetraRh.(1) At various timepoints, the cells were fixed and analysed via flow cytometry. Negative controls consisted of *Msm* treated with meropenem-clavulanic acid (100 µg/mL). Error bars represent standard deviation from the mean (n = 3).

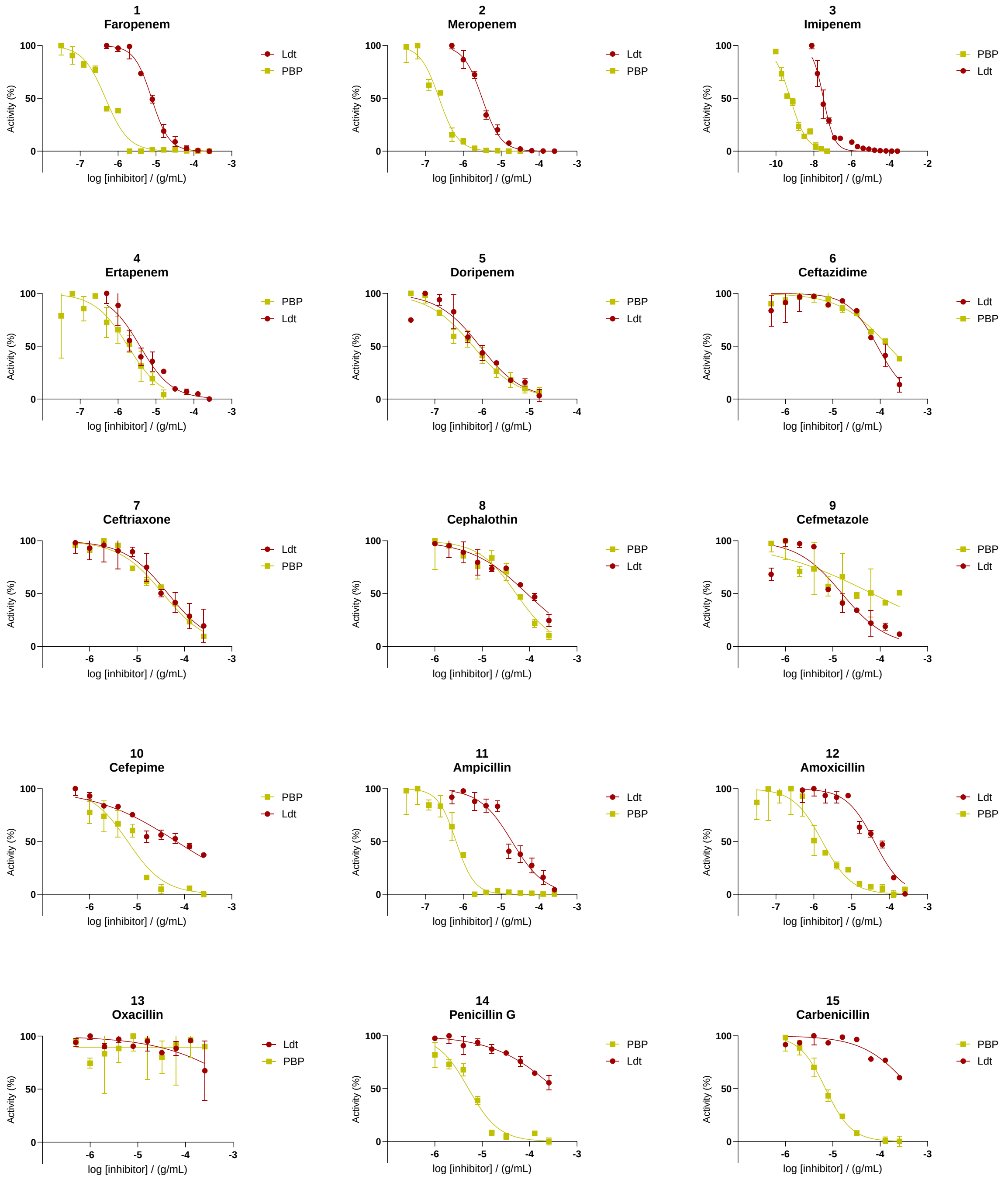

Figure S4. Inhibition of penicillin binding proteins and L,D-transpeptidases of *Mycobacterium smegmatis*. [continues]

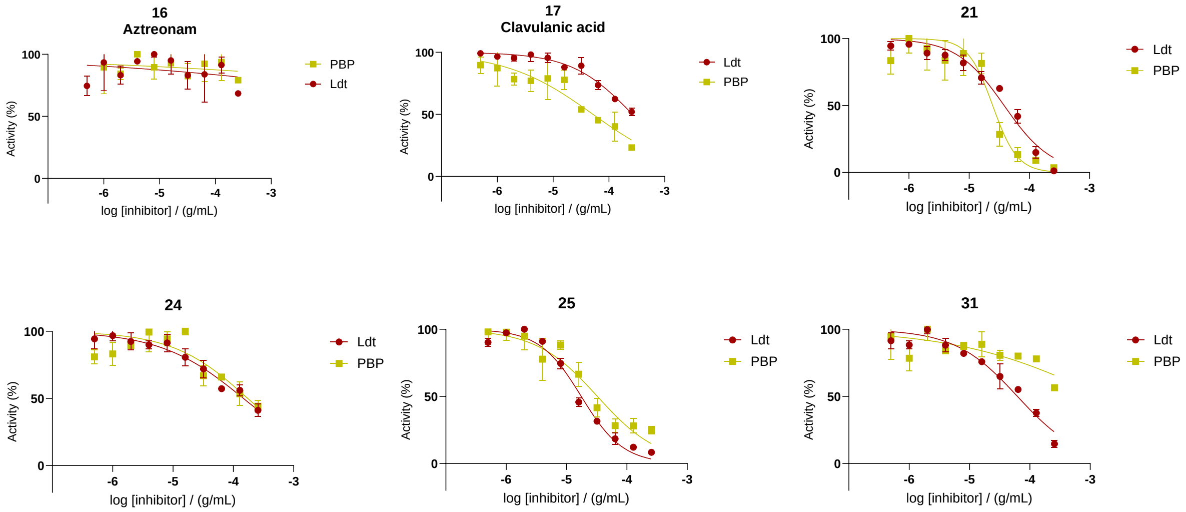

Figure S4. Inhibition of penicillin binding proteins and L,D-transpeptidases of *Mycobacterium smegmatis*. A culture of *Msm* (OD_{600} 0.8) was incubated with 50 µM PentaFl, or 0.5 µM TetraRh in the presence of various concentrations of inhibitors, and incubated for 24 hours at 37 °C, then fixed and analysed by flow cytometry. In the case of ertapenem, doripenem, ceftazidime, ceftriaxone, cephalothin, cefmetazole, cefepime, ampicillin, amoxicillin, oxacillin, penicillin G, carbenicillin, and aztreonam, assays were performed in the presence of 100 µg/mL clavulanic acid. Error bars represent standard deviation from the mean (n = 2). The pIC_{50} values and compound structures are given in Table S1.

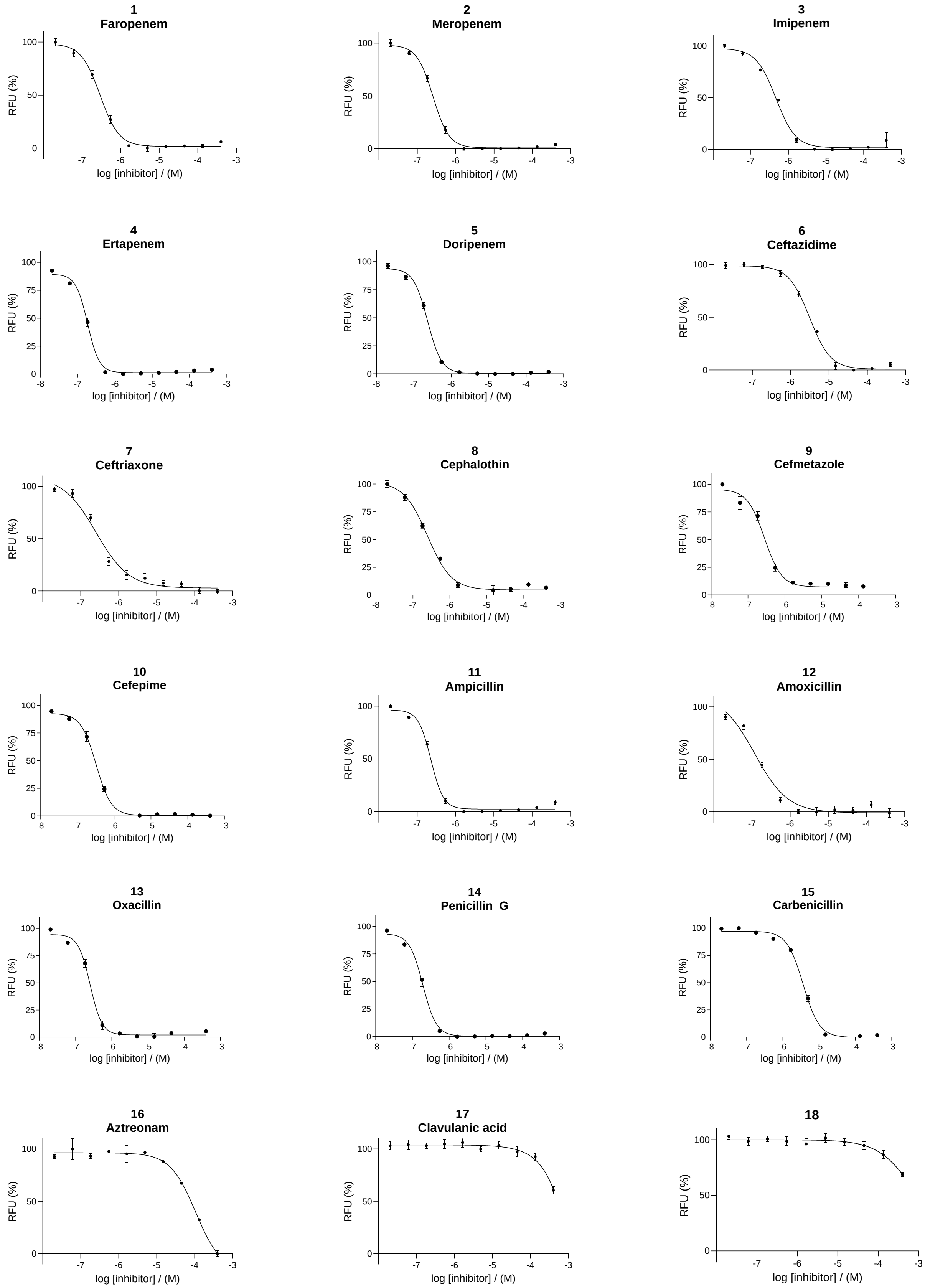

Figure S5. Inhibition of PBP3 of *Mycobacterium tuberculosis*. [Continues]

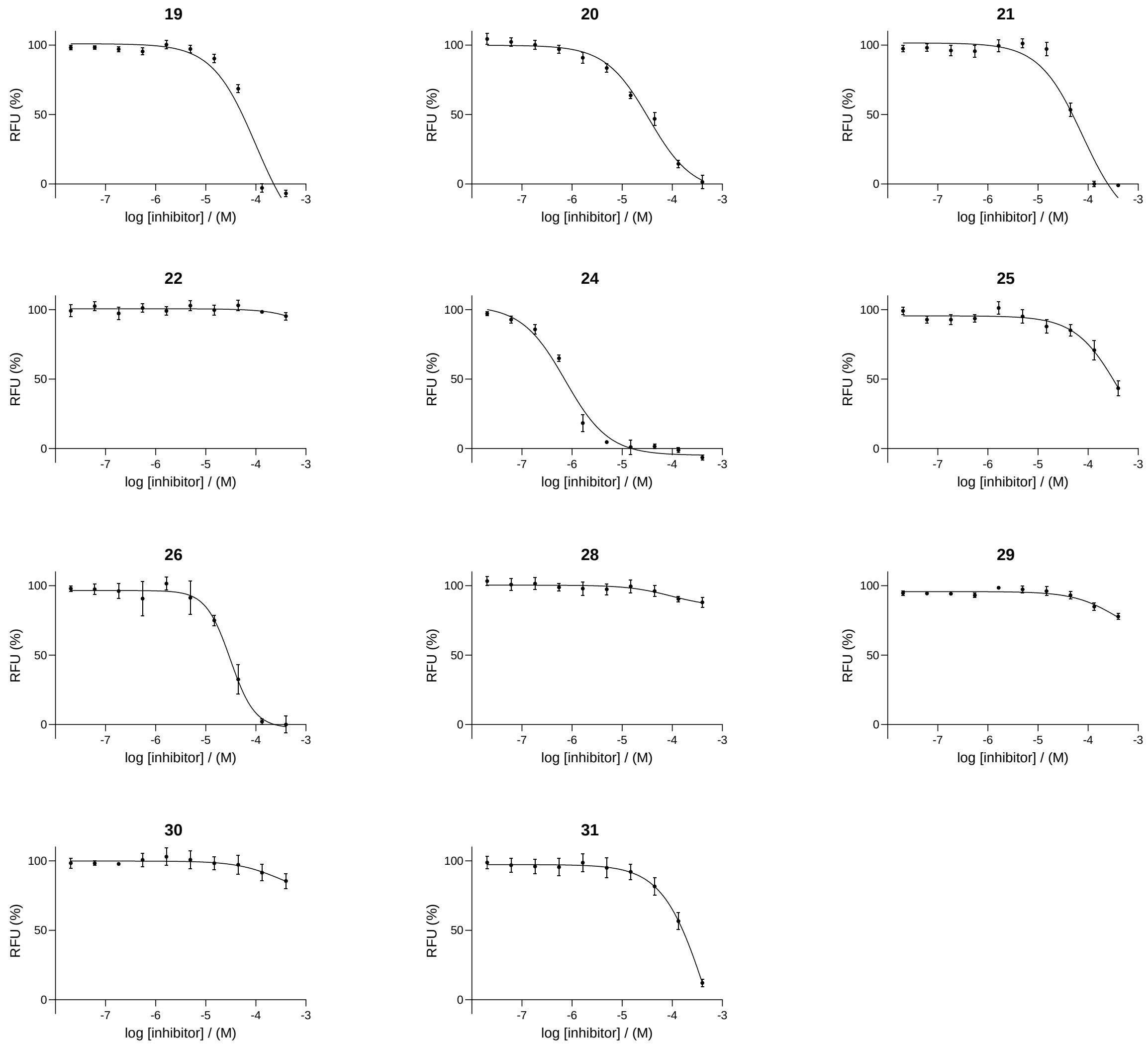

Figure S5. Inhibition of PBP3 of *Mycobacterium tuberculosis* by 1-31. PBP3 (300 nM) was incubated with the specified inhibitor for 10 minutes at room temperature. Activities were assessed with S2d (1.5 mM), monobromobimane (mBBr; 0.05 mM), and D-Ala (1 mM) in 50 mM HEPES pH 7.4, 100 mM NaCl, 0.01% (*v*/*v*) Triton X-100. Error bars represent standard deviation from the mean (n = 4). The pIC_{50} values and compound structures are given in Table S1.

Table S5. Structure-activity relationship studies of sulfonyl pyridines for inhibition of *Mycobacterium smegmatis* and reactivity with Ldt_{Mt2} and glutathione. The calculated modified Ldt_{Mt2} mass corresponds to the mass resulting from an aromatic nucleophilic substitution (S_NAr) reaction (+0 Da = unmodified Ldt_{Mt2}). MIC values were determined in triplicate assays, Ldt_{Mt2} and GSH reactivity and inhibition assays were determined in quadruplicate assays. Errors represent standard deviation from the mean. See Methods for experimental details. All mass shifts were consistent with S_NAr reaction of the assigned structures of the tested compounds.

Compound	Structure	*Msm* MIC (µg/mL)	pIC_{50} Ldt_{Mt2}	k_{inact}/K_I Ldt_{Mt2} (M^{-1} s^{-1})	k_{chem} (M^{-1} s^{-1})	Ldt_{Mt2} calculated mass (Da)	Ldt_{Mt2} observed mass (Da)
**31**	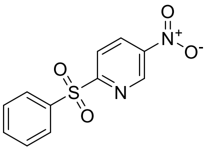	8	5.3 ± 0.12	52.6 ± 0.62	59.7 ± 2.6	38101 (+123)	38101 (+123)
**32**	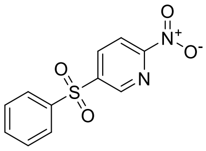	4	4.6 ± 0.34	<10.0	<0.83	38101 (+123)	38196 (+218)
**33**	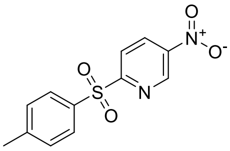	8	5.8 ± 0.15	51.9 ± 2.5	95.8 ± 1.0	38101 (+123)	38101 (+123)
**34**	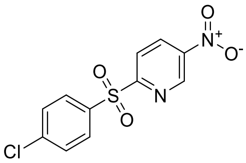	4	5.6 ± 0.15	86.9 ± 1.7	85.9 ± 7.2	38101 (+123)	38101 (+123)
**35**	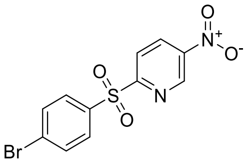	8	6.1 ± 0.12	272 ± 1.7	96.6 ± 1.5	38101 (+123)	38101 (+123)
**36**	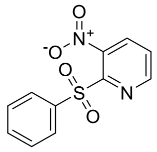	16	4.6 ± 0.26	<10.0	22.6 ± 3.6	38101 (+123)	38101 (+123)
**37**	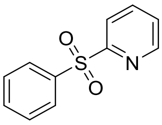	>128	<4.4	<10.0	<0.83	38056 (+78)	37978 (+0)
**38**	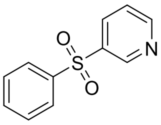	>128	<4.4	<10.0	<0.83	38056 (+78)	37978 (+0)

Compound	Structure	*Msm* MIC (µg/mL)	pIC_{50} Ldt_{Mt2}	k_{inact}/K_I Ldt_{Mt2} (M^{-1} s^{-1})	k_{chem} (M^{-1} s^{-1})	Ldt_{Mt2} calculated mass (Da)	Ldt_{Mt2} observed mass (Da)
**39**	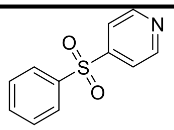	>128	<4.4	<10.0	<0.83	38056 (+78)	37978 (+0)
**40**	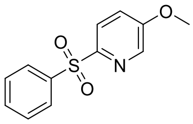	>128	<4.4	<10.0	<0.83	38086 (+108)	37978 (+0)
**41**	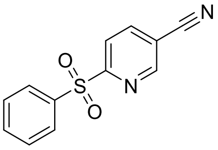	>128	4.6 ± 0.36	<10.0	3.2 ± 0.73	38081 (+103)	38081 (+103)
**42**	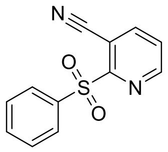	>128	<4.4	<10.0	<0.83	38081 (+103)	38101 (+123), 38222 (+244), 38345 (+367)
**43**	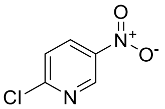	32	<4.4	<10.0	<0.83	38101 (+123)	37978 (+0)
**44**	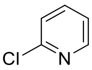	>128	<4.4	<10.0	<0.83	38056 (+78)	37978 (+0)
**45**	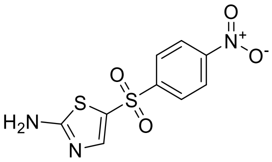	>128	N.D.	N.D.	N.D.	38100 (+122)	38132 (+154)
**46**	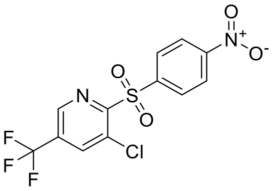	>128	<4.4	<10.0	<0.83	38158 (+180)	38158 (+180)

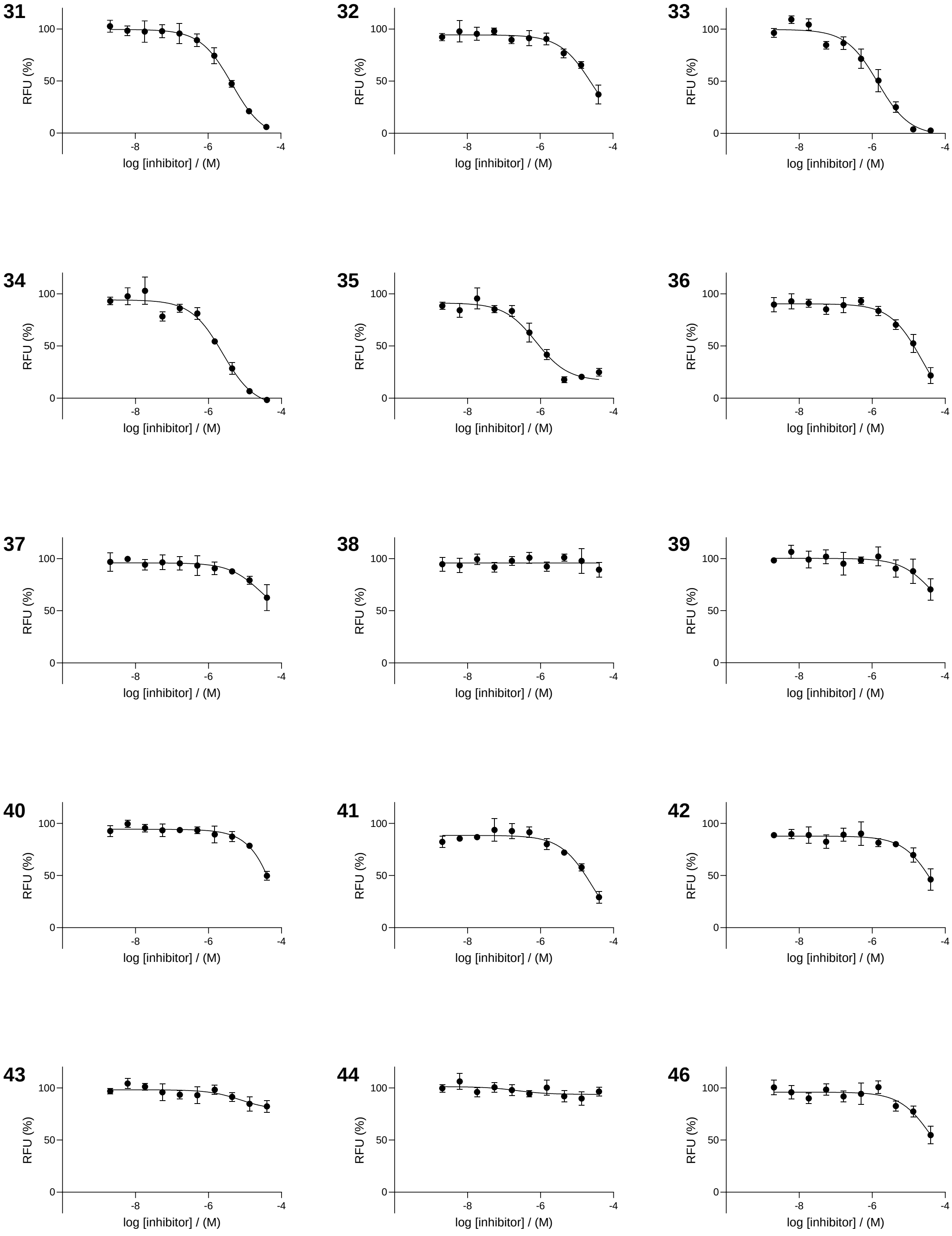

Figure S6. Dose-response curves for sulfonyl pyridines 31-46 with Ldt_{Mt2}. Inhibition assays were carried out using 100 nM Ldt_{Mt2} and 25 μM Probe 1 with 10 min pre-incubation at room temperature in 50 mM HEPES, pH 7.2 with 0.01% (*v/v*) Triton X-100. Error bars represent standard deviations from the mean (n = 4). Average pIC_{50} values and compound structures are given in Table S5.

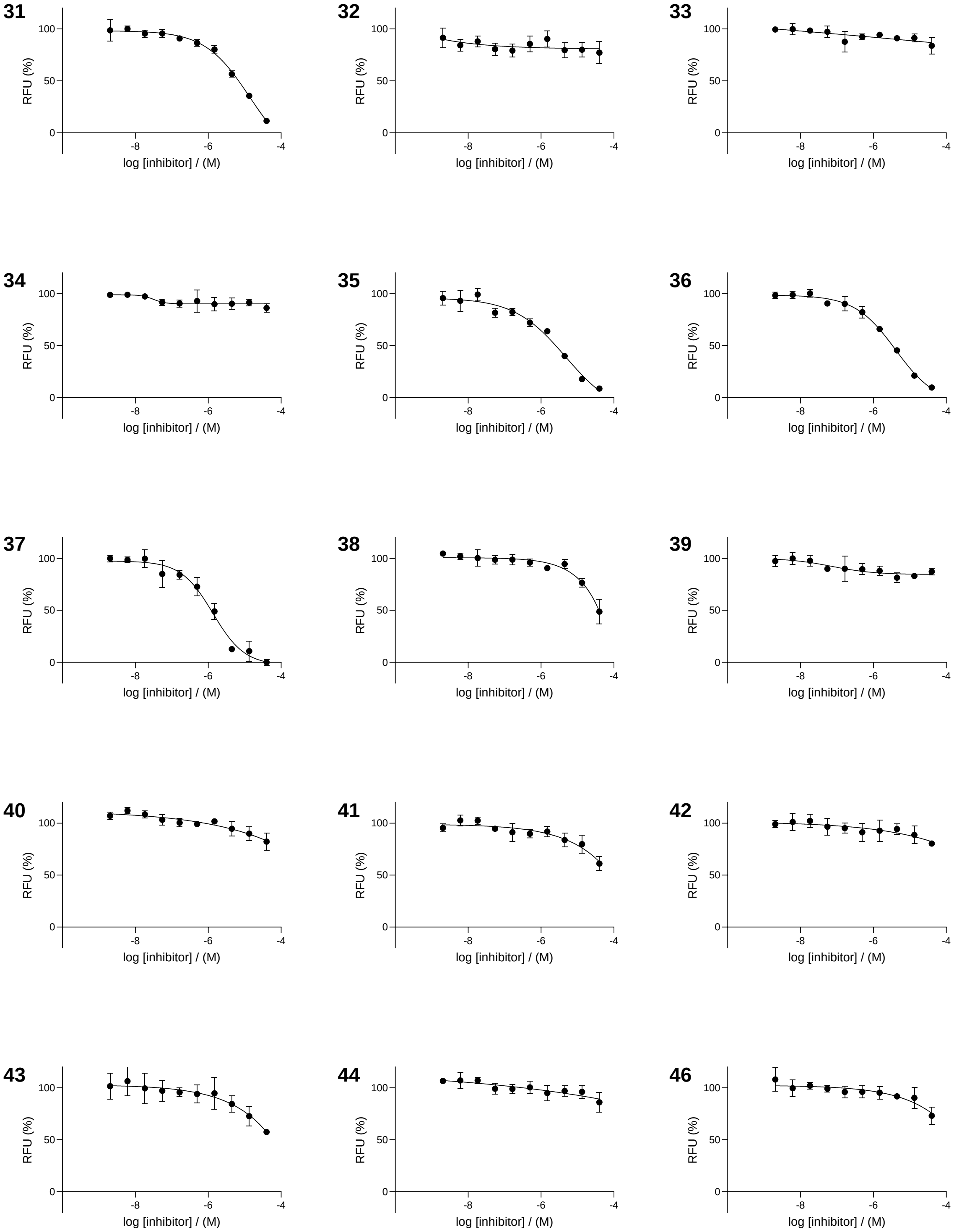

Figure S7. Determination of the second-order rate constant for irreversible inhibition (k_{inact}/K_I) of Ldt$_{Mt2}$ by sulfonyl pyridines 31-46. Inhibition assays were carried out using 100 nM Ldt$_{Mt2}$ and 10 μM Probe 1,(2) with 3 h incubation at rt in 50 mM HEPES, pH 7.2 with 0.01% (*v/v*) Triton X-100. Error bars represent standard deviations from the mean (n = 4). Average (k_{inact}/K_I)$_{inhibitor}$ values and compound structures are given in Table S5.

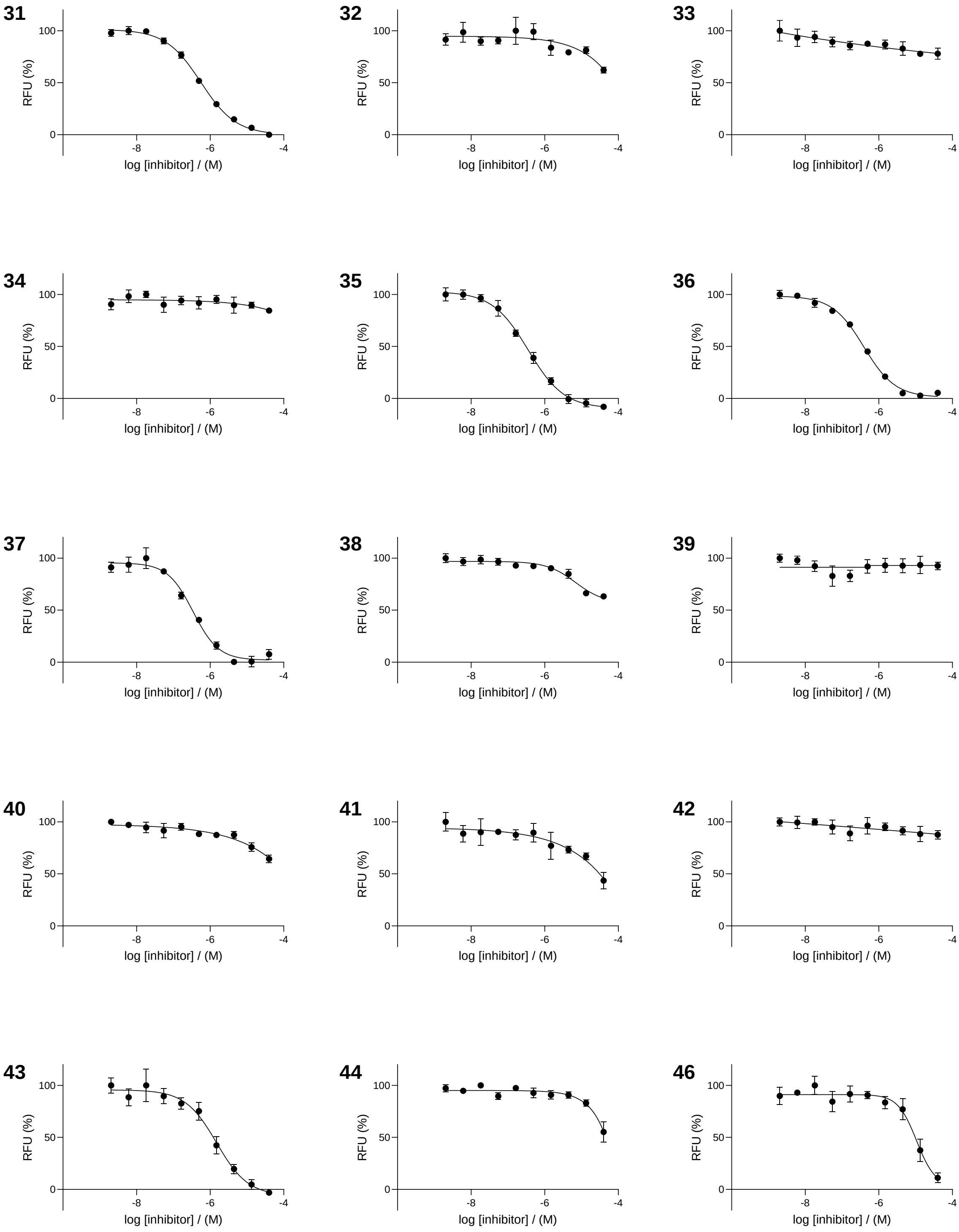

Figure S8. Determination of the intrinsic thiol reactivity rate constant (k_{chem}) for sulfonyl pyridines 31-46. Assays were carried out using 500 nM L-glutathione and 10 µM Probe 1 with 16 h incubation at room temperature in 50 mM HEPES, pH 7.2 with 0.01% (*v/v*) Triton X-100. Error bars represent standard deviations from the mean (n = 4). Average k_{chem} values and compound structures are given in Table S5.

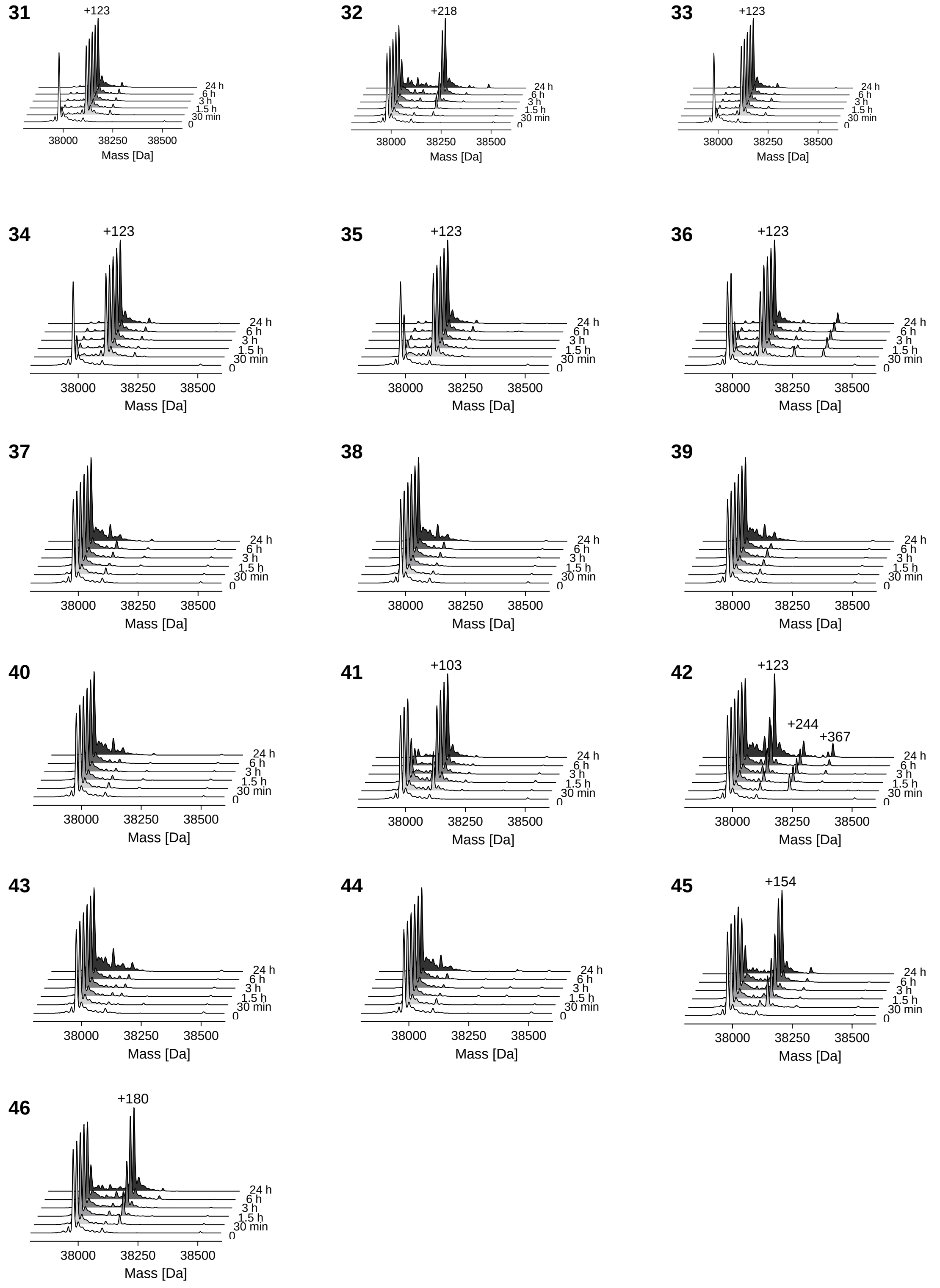

Figure S9. Protein observed SPE-MS analysis for the reaction of sulfonyl pyridines 31-46 with Ldt_{Mt2}. Ldt_{Mt2} (1 µM) was incubated with the specified inhibitor (100 µM) at rt in 50 mM Tris, pH 7.5. Samples were analysed after the indicated times. Deconvoluted mass spectra, obtained using the maximum entropy algorithm in the MassHunter Workstation Qualitative Analysis B.07.00 program (Agilent), are shown. Mass shifts and assignments are given in Table S5. All mass shifts were consistent with $\text{S}_{\text{N}}\text{Ar}$ reaction of the assigned structures of the tested compounds.

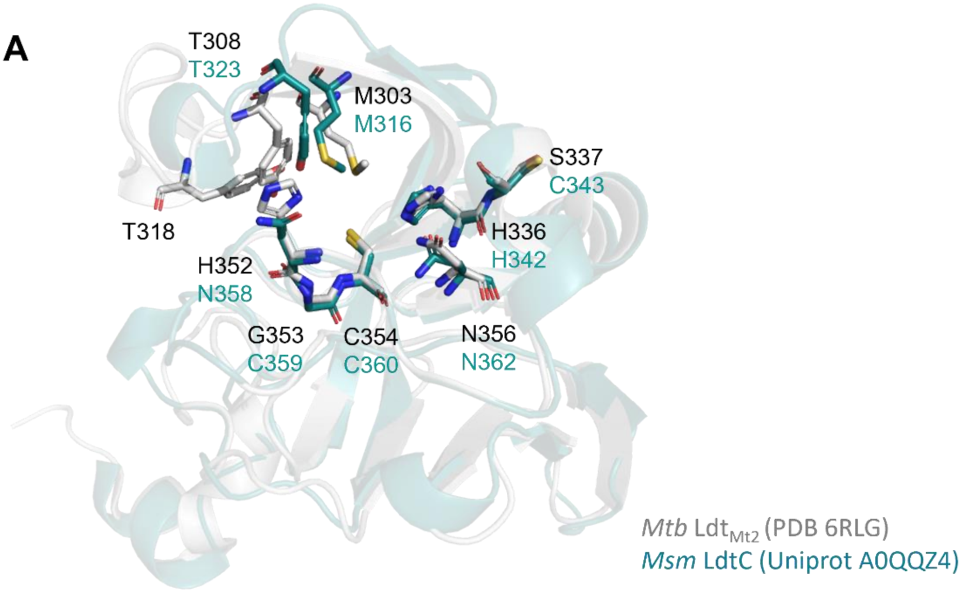

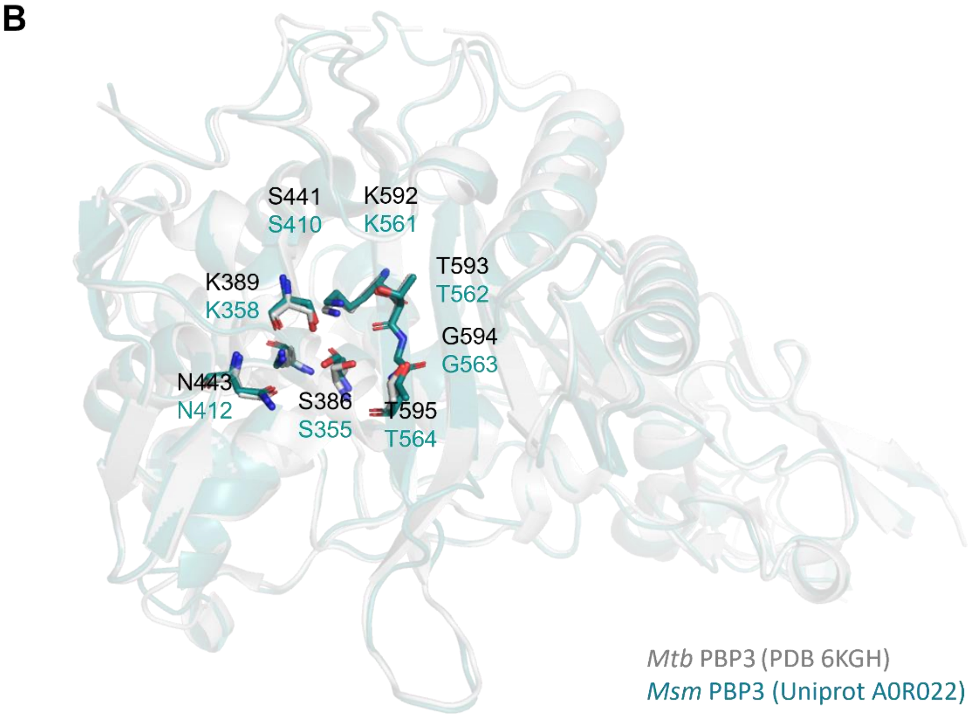

Figure S10. Structural comparison between the essential Ldt and PBP from *Mycobacterium tuberculosis* and *Mycobacterium smegmatis*. A. Comparison of the catalytic domain of Ldt$_{Mt2}$ of *Mtb* (PDB 6RLG; grey)(6) and the catalytic domain of LdtC of *Msm* (Uniprot A0QQZ4, as predicted with AlphaFold; teal)(7). **B**. Comparison of the catalytic domain of PBP3 of *Mtb* (PDB 6KGH)(8) and of *Msm* (Uniprot A0R022, as predicted with AlphaFold; teal)(7).

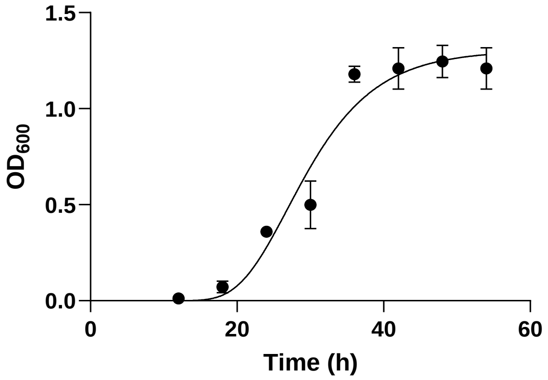

Figure S11. Growth curve of *Mycobacterium smegmatis*. A culture of *Msm* was grown in Middlebrook 7H9 broth supplemented with 0.1% (*v*/*v*) Tween-80, 0.5 % (*w*/*v*) bovine albumin fraction V, 0.2% (*w*/*v*) dextrose, and 0.3 % (*v*/*v*) catalase (beef) in 37 °C with aeration following inoculation (0.1%). Growth was assessed with via measurement of OD_{600}. After a lag phase of ~12 h, the *Msm* culture was observed to reach the exponential growth phase with a generation time of 3.6 h, following which time the stationary phase was reached at ~42 h.

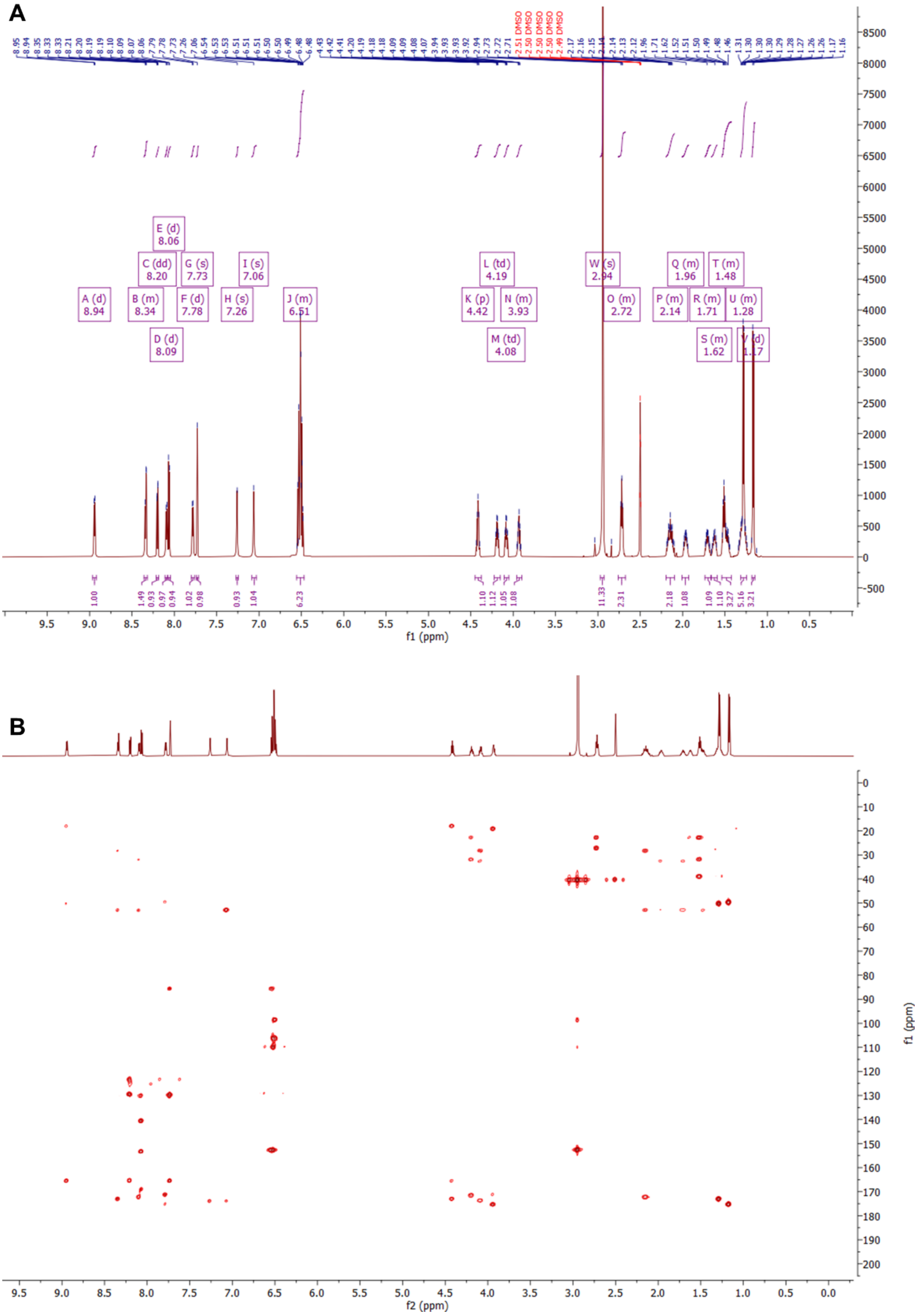

Figure S12. Characterisation of TetraRh by ^{1}H (700 MHz) and two-dimensional NMR experiments. [continues]

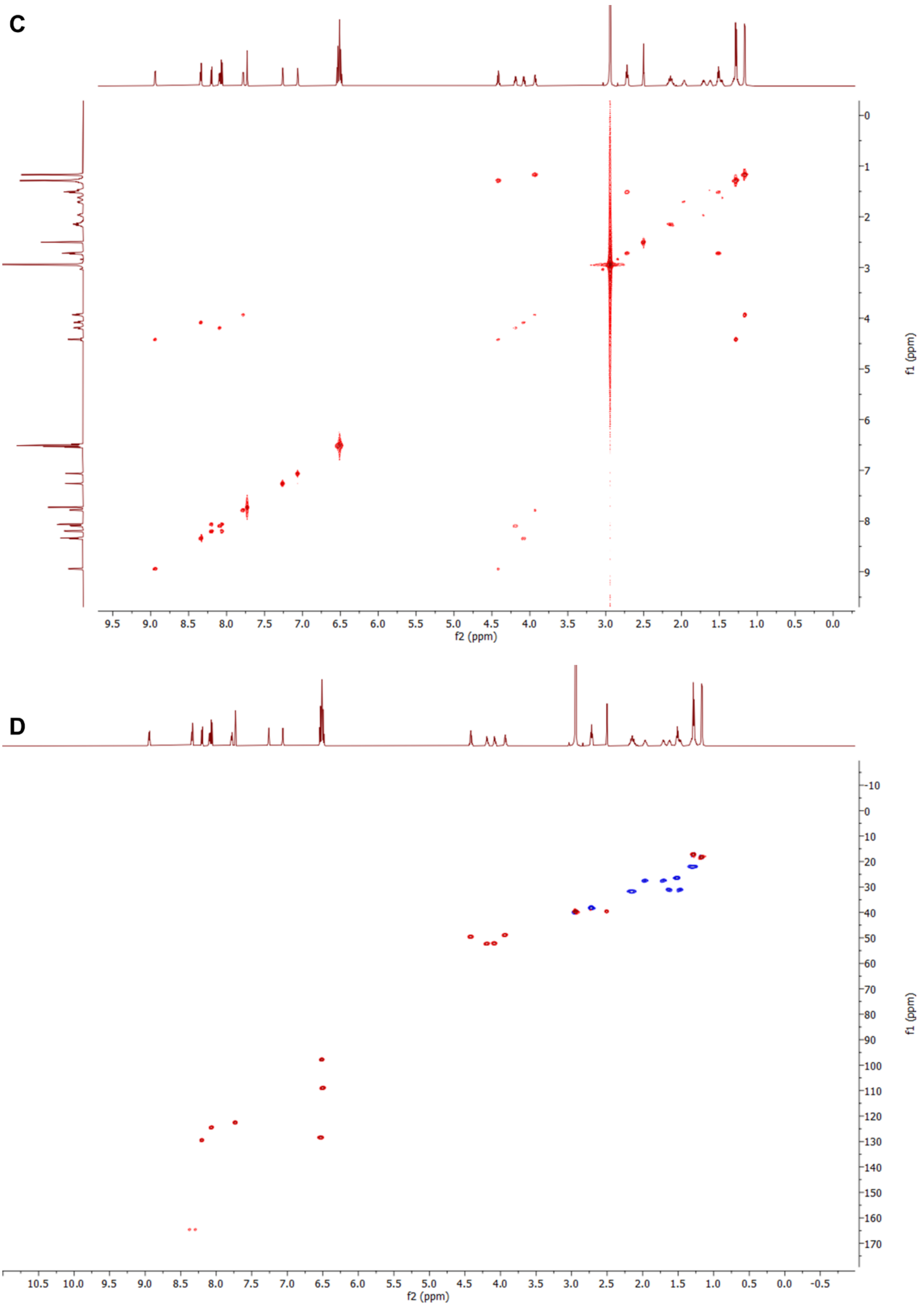

Figure S12. Characterisation of TetraRh by ^{1}H (700 MHz) and two-dimensional NMR experiments. NMR analysis of TetraRh comprised of ^{1}H (**A**), ^{1}H,^{13}C-HMBC (**B**), ^{1}H,^{1}H-COSY (**C**), and ^{1}H,^{13}C-HSQC (**D**). Assignments of the ^{1}H chemical shifts are given in Table S6.

Table S6. Assignment of ^{1}H-NMR chemical shifts (ppm) for TetraRh. Assignments were based on the spectra shown in Figure S12.

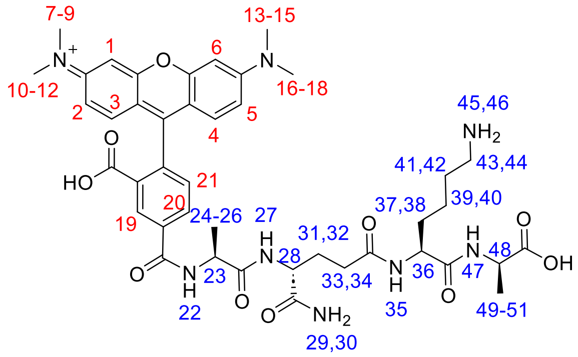

TetraRh

Chemical shift (ppm)	Multiplicity	Integration	Assignment
8.94	d, *J* = 6.5 Hz	1 H	47
8.36 – 8.32	m	1 H	27
8.2	dd, *J* = 8.1, 1.4 Hz	1 H	20
8.09	d, *J* = 8.2 Hz	1 H	35
8.06	d, *J* = 8.1 Hz	1 H	21
7.78	d, *J* = 6.9 Hz	1 H	22
7.73	s	1 H	19
7.26	s	1 H	29
7.06	s	1 H	30
6.56 – 6.47	m	6 H	1-6
4.42	p, *J* = 7.0 Hz	1 H	48
4.19	td, *J* = 8.4, 5.2 Hz	1 H	36
4.08	td, *J* = 8.6, 4.9 Hz	1 H	28
3.96 – 3.90	m	1 H	23
3.94	s	12 H	7-18
2.76 – 2.68	m	2 H	43, 44
2.19 – 2.10	m	2 H	33, 34
2.01 – 1.93	m	1 H	31
1.74 – 1.67	m	2 H	37, 32
1.54 – 1.43	m	3 H	38, 39, 40
1.32 – 1.25	m	5 H	49-51, 41, 42
1.17	d, *J* = 7.1 Hz	3 H	24-26

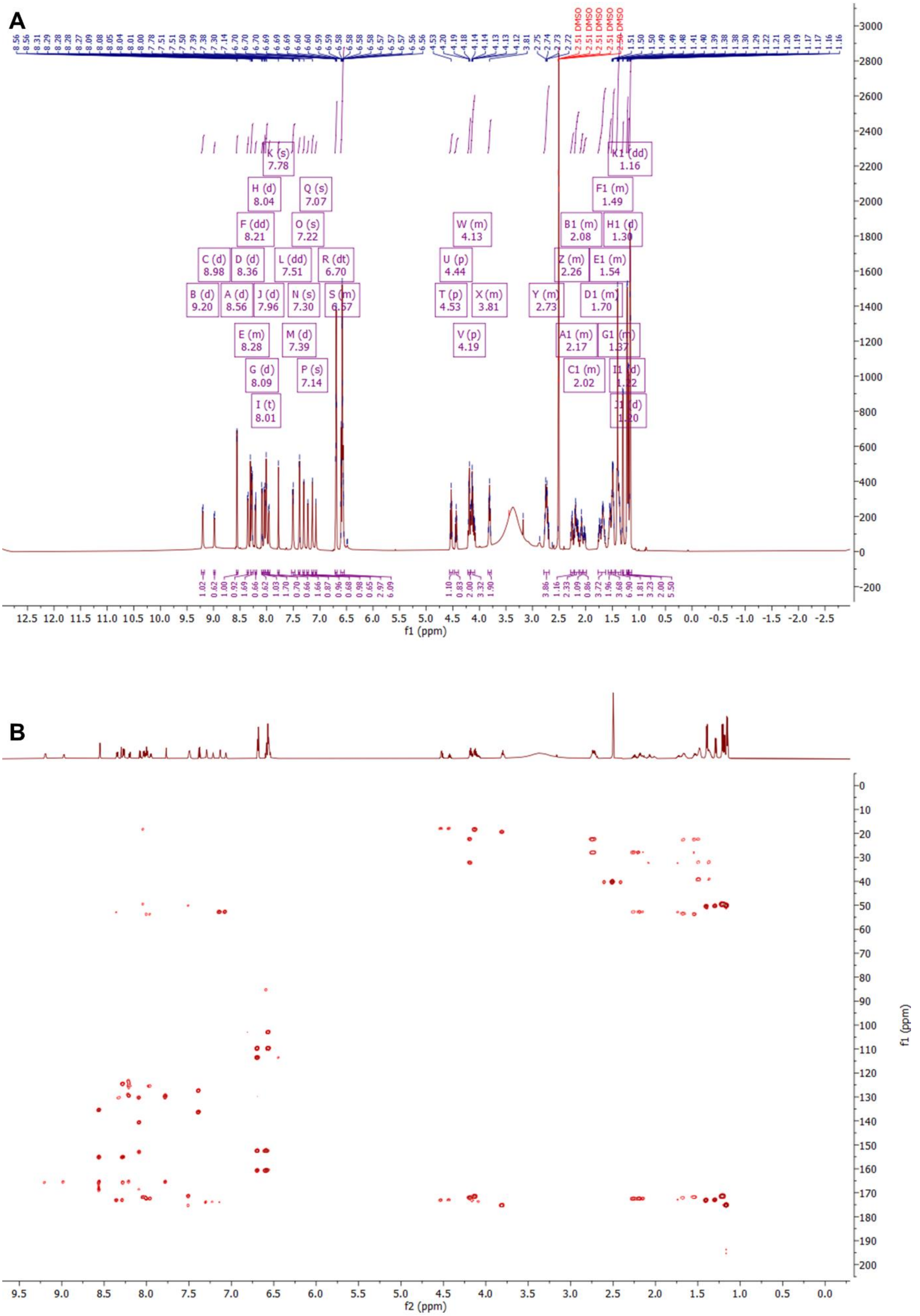

Figure S13. Characterisation of PentaFl by ^{1}H (700 MHz) and two-dimensional NMR experiments. [continues]

C

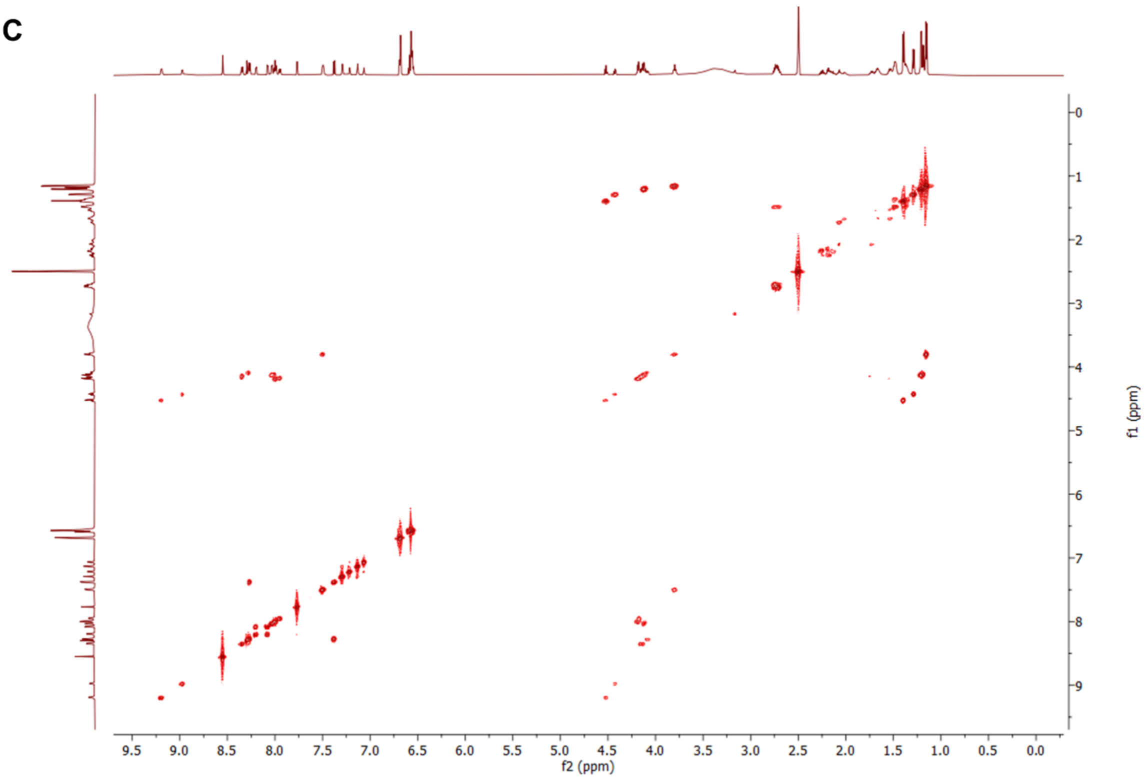

D

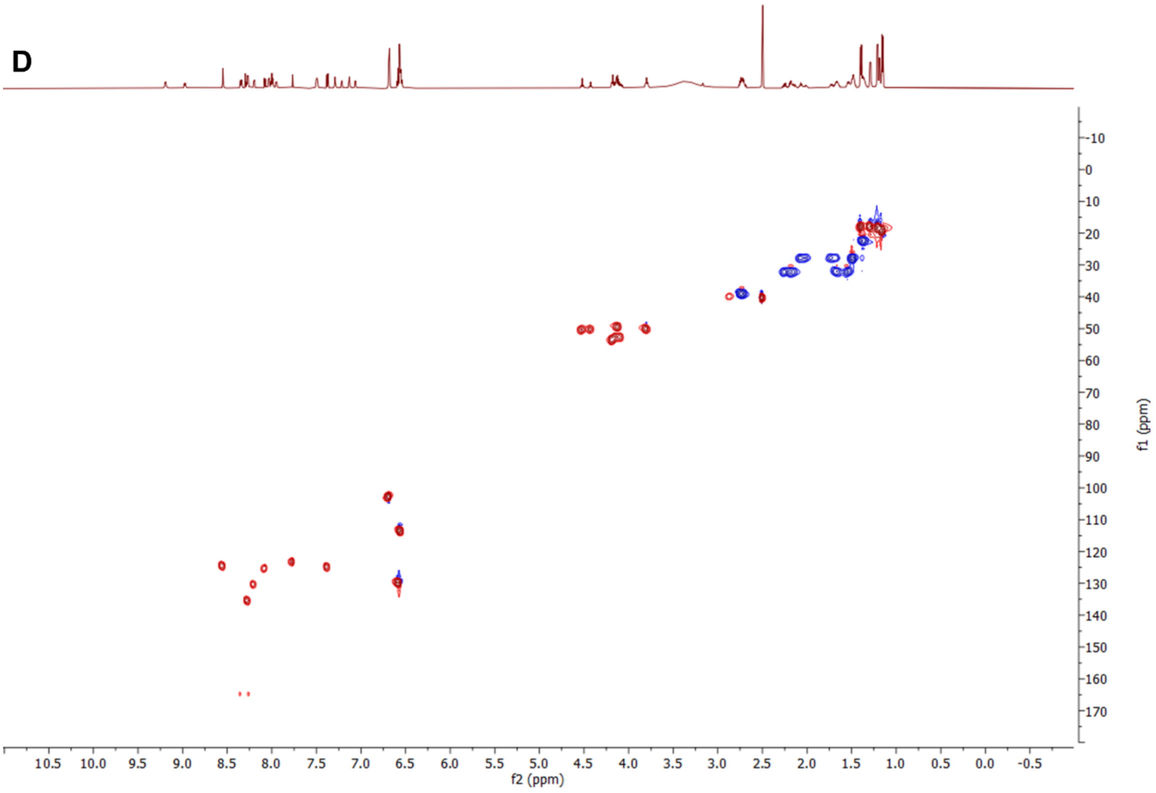

Figure S13. Characterisation of PentaFI by ^{1}H (700 MHz) and two-dimensional NMR experiments. NMR analysis of PentaFI comprised of ^{1}H (**A**), ^{1}H,^{13}C-HMBC (**B**), ^{1}H,^{1}H-COSY (**C**), and ^{1}H,^{13}C-HSQC (**D**). Assignment of the ^{1}H chemical shifts are given in Table S7. Note that PentaFI exists as two isomers (isomer 1:isomer 2 ratio 1:0.7, respectively; see Table S7).

Table S7. Assignment of ^{1}H-NMR chemical shifts (ppm) for PentaFl. Assignments were based on the spectra shown in Figure S13. Note that PentaFl exists as two isomers (isomer 1:isomer 2 ratio 1:0.7, respectively).

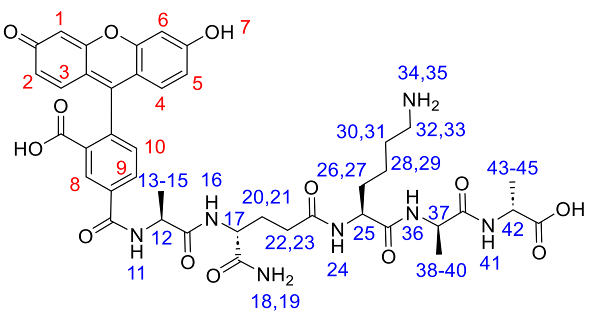

PentaFl, isomer 1

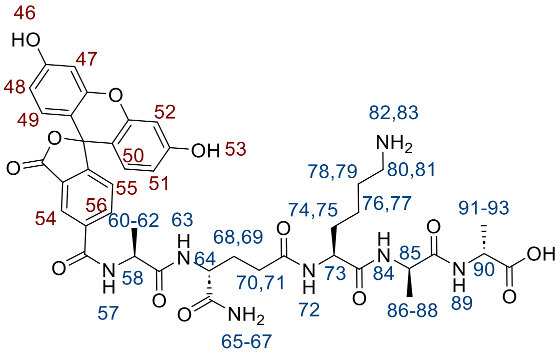

PentaFl, isomer 2

Chemical shift (ppm)	Multiplicity	Integration	Assignment
9.20	d, *J* = 6.7 Hz	1 H	9
8.98	d, *J* = 6.6 Hz	0.7 H	1-6, 47-51
8.56	s	1 H	8
8.36	d, *J* = 8.1 Hz	1 H	7
8.30 – 8.26	m	1.7 H	10, 53, 46
8.21	dd, *J* = 8.0, 1.4 Hz	0.7 H	56
8.09	d, *J* = 8.1 Hz	0.7 H	55
8.04	d, *J* = 7.6 Hz	1 H	NH
8.01	t, *J* = 7.8 Hz	1.7 H	NH
7.96	d, *J* = 7.7 Hz	0.7 H	NH
7.77	s	0.7 H	54
7.51	dd, *J* = 6.5, 3.0 Hz	1.7 H	1-6, 47-51
7.39	d, *J* = 8.0 Hz	1 H	1-6, 47-51
7.30	s	1 H	NH
7.22	s	0.7 H	NH
7.14	s	1 H	NH
7.07	s	0.7 H	NH
6.70	dt, *J* = 6.8, 2.4 Hz	3 H	1-6, 47-51
6.61 – 6.54	m	6 H	1-6, 47-51
4.53	p, *J* = 7.1 Hz	1 H	42
4.44	p, *J* = 7.0 Hz	0.7 H	90
4.19	p, *J* = 6.6 Hz	2 H	64, 73
4.17 – 4.07	m	3 H	37, 17, 85
3.83 – 3.78	m	2 H	12, 58
2.79 – 2.69	m	3.7 H	32, 33, 80, 81
2.29 – 2.22	m	1.2 H	70, 71
2.21 – 2.13	m	2 H	22, 23
2.11 – 2.05	m	1 H	21
2.05 – 1.99	m	0.7 H	69
1.78 – 1.62	m	3.7 H	26, 27, 74, 75
1.58 – 1.52	m	2 H	20, 68
1.52 – 1.46	m	3.7 H	30, 31, 78, 79
1.44 – 1.34	m	7 H	43-45, 28, 29, 76, 77
1.30	d, *J* = 7.1 Hz	2 H	91-93
1.22	d, *J* = 7.3 Hz	3 H	38-40
1.20	d, *J* = 7.3 Hz	2 H	86-88
1.16	dd, *J* = 7.0, 2.0 Hz	5 H	13-15, 60-62

References

1. Pidgeon SE, Apostolos AJ, Nelson JM, Shaku M, Rimal B, Islam MN, Crick DC, Kim SJ, Pavelka MS, Kana BD, Pires MM. 2019. L,D-Transpeptidase Specific Probe Reveals Spatial Activity of Peptidoglycan Cross-Linking. ACS Chem Biol 14:2185–2196.
2. de Munnik M, Lohans CT, Langley GW, Bon C, Brem J, Schofield CJ. 2020. A Fluorescence-Based Assay for Screening β-Lactams Targeting the *Mycobacterium tuberculosis* Transpeptidase Ldt_{Mt2}. ChemBioChem 21:368-372.
3. de Munnik M, Lang PA, De Dios Antos F, Cacho M, Bates RH, Brem J, Rodríguez-Miquel B, Schofield CJ. 2023. High-Throughput Screen with the L,D-transpeptidase Ldt_{Mt2} of *Mycobacterium tuberculosis* Reveals Novel Classes of Covalently Reacting Inhibitors Chem Sci 14:7262-7278.
4. Lelovic N, Mitachi K, Yang J, Lemieux MR, Ji Y, Kurosu M. 2020. Application of Mycobacterium smegmatis as a surrogate to evaluate drug leads against Mycobacterium tuberculosis. J Antibiot 73:780-789.
5. Agrawal P, Miryala S, Varshney U. 2015. Use of Mycobacterium smegmatis Deficient in ADP-Ribosyltransferase as Surrogate for Mycobacterium tuberculosis in Drug Testing and Mutation Analysis. PloS one 10:e0122076.
6. de Munnik M, Lohans CT, Lang PA, Langley GW, Malla TR, Tumber A, Schofield CJ, Brem J. 2019. Targeting the *Mycobacterium tuberculosis* transpeptidase Ldt_{Mt2} with cysteine-reactive inhibitors including ebselen. Chem Commun 55:10214-10217.
7. Jumper J, Evans R, Pritzel A, Green T, Figurnov M, Ronneberger O, Tunyasuvunakool K, Bates R, Žídek A, Potapenko A, Bridgland A, Meyer C, Kohl SAA, Ballard AJ, Cowie A, Romera-Paredes B, Nikolov S, Jain R, Adler J, Back T, Petersen S, Reiman D, Clancy E, Zielinski M, Steinegger M, Pacholska M, Berghammer T, Bodenstein S, Silver D, Vinyals O, Senior AW, Kavukcuoglu K, Kohli P, Hassabis D. 2021. Highly accurate protein structure prediction with AlphaFold. Nature 596:583-589.
8. Lu Z, Wang H, Zhang A, Liu X, Zhou W, Yang C, Guddat L, Yang H, Schofield CJ, Rao Z. 2020. Structures of *Mycobacterium tuberculosis* Penicillin-Binding Protein 3 in Complex with Five β-Lactam Antibiotics Reveal Mechanism of Inactivation. Mol Pharmacol 97:287-294.
